# Biodegradable Prussian blue/manganese dioxide core–shell nanoparticles with open cages for imaging-guided chemo-photothermal combined therapy of cancer cells

**DOI:** 10.1039/d5ra07493b

**Published:** 2026-02-23

**Authors:** Ying Gao, Jinbo Xue, Yuebo Yang, Dongxiao Bian, Luyao Liu, Ming Zhu, Tao Yang, Le Liu

**Affiliations:** a Department of Stomatology, The 964th Hospital of the Chinese People’s Liberation Army Joint Logistics Support Force Changchun Jilin China 1595765865@qq.com 1350288208@qq.com; b Faculty of Education, The University of Hong Kong Hong Kong

## Abstract

Imaging-guided diagnostic and therapeutic strategies have attracted significant attention in the field of nanomedicine. To develop more efficient theranostic nanoplatforms, this study designed and synthesized Prussian blue (PB)/manganese dioxide (MnO_2_) core–shell nanoparticles with an open cage-like nanostructure (denoted as PBMn core–shell nanoparticles). In this nanoplatform, the PB core serves as a photothermal agent for near-infrared laser-induced photothermal therapy (PTT) against tumor cells, owing to its strong absorption in the NIR region and excellent stability. The MnO_2_ shell, formed *via* the reduction of KMnO_4_ by glucose, not only effectively modulates the size and optical properties of the PB core but also significantly depletes the intracellular glutathione (GSH) level in tumor cells. Moreover, the Mn^2+^ generated from the reduction of MnO_2_ by highly concentrated GSH in the tumor microenvironment synergistically enhance both *T*_1_- and *T*_2_-weighted magnetic resonance imaging (MRI) signals, enabling high-contrast tumor imaging. Based on these features, the PBMn core–shell nanoparticles have been successfully applied for MRI-guided chemo-photothermal combination cancer therapy.

## Abstract 2

The open cage-like Prussian blue/manganese dioxide (PBMn) core–shell nanoplatform utilizes its PB core for photothermal therapy and its MnO_2_ shell to enhance MRI, enabling imaging-guided synergistic chemo-photothermal therapy.

## Graphical abstract

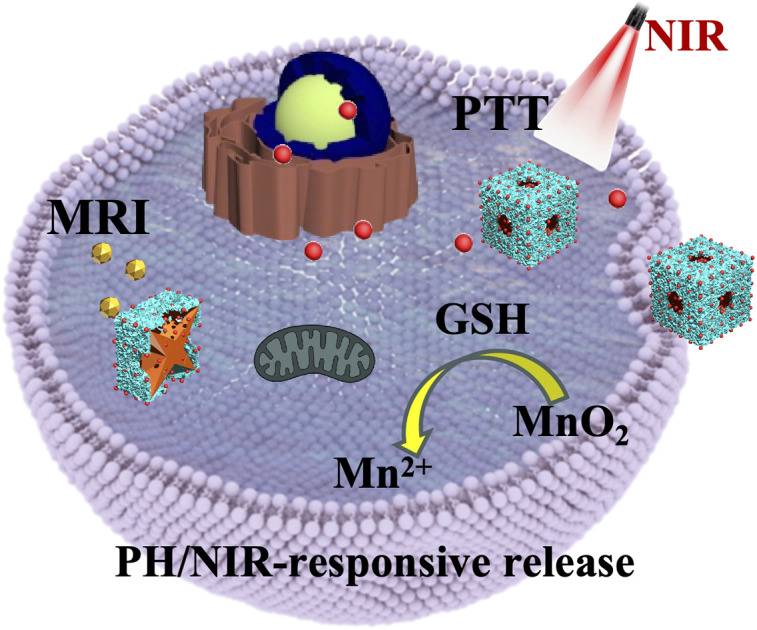

## 1 Introduction

Advances in nanotechnology and nanobiomaterials have opened up broad prospects for cancer diagnosis and treatment.^1,2^ Owing to their ease of multifunctional modification, nanoparticles (NPs) have been extensively designed for simultaneous cancer detection and therapy, promoting the rapid development of multifunctional nanostructures.^3,4^ Compared to traditional radiotherapy and chemotherapy, photothermal therapy (PTT) offers distinct therapeutic advantages by efficiently ablating cancer cells while causing significantly less damage to normal tissues.^5,6^ Up to now, a variety of photothermal materials with strong absorption in the near-infrared (NIR) region have been developed and applied in PTT research, such as various Au nanostructures, carbon nanomaterials, CuS NPs, Prussian blue (PB) and others.^7,8^ Among these NIR absorbing nanomaterials, PB NPs stand out due to their excellent NIR absorption, high photothermal conversion efficiency, and good biocompatibility, with their antitumor efficacy having been confirmed by numerous studies.^9,10^ Furthermore, research has shown that combining photothermal therapy with chemotherapy can significantly enhance antitumor outcomes compared to either treatment modality alone.^11,12^ However, the inherent small pore size and limited drug-loading capacity of PB restrict its application as a drug delivery system in combination therapy. To address this issue, researchers have attempted to construct porous shells on the surface of PB to enable the loading of anticancer drugs, thereby introducing chemotherapeutic functionality. For example, Su *et al.* developed a mesoporous silica-coated PB nanoplatform for chemo-photothermal combination therapy.^13^ On the other hand, PB NPs, owing to their high iron content (particularly Fe^3+^), possess potential as contrast agents for magnetic resonance imaging (MRI).^14,15^ MRI provides the benefit of high soft-tissue contrast and anatomical imaging without ionizing radiation, enabling precise visualization of tumors for guided therapy.^16,17^ Nevertheless, their diagnostic performance, especially longitudinal relaxivity, remains suboptimal.^18^ In recent years, manganese dioxide (MnO_2_) nanostructures have garnered widespread attention in the field of cancer theranostics.^19^ Studies have demonstrated that MnO_2_ can decompose upon reaction with overexpressed glutathione (GSH) or H^+^ in the tumor microenvironment, releasing Mn^2+^ that effectively enhance *T*_1_-weighted MRI contrast, significantly improving tumor imaging clarity.^20,21^ Moreover, the Mn^2+^ generated in this process are water soluble and biocompatible, and can be rapidly cleared by the kidneys, avoiding the long term toxicity concerns associated with non-biodegradable inorganic nanomaterials and endowing MnO_2_ with notable advantages for *in vivo* applications.^22^ Inspired by the advantageous performance of MnO_2_ in enhancing MRI, nanocomposites combining MnO_2_ with PB have attracted considerable attention. For example, Peng *et al.* successfully synthesized PB/MnO_2_ hybrid nanocrystals and applied them to trimodal imaging guided photothermal therapy for breast cancer.^23^ However, PB and its analogues are excellent multifunctional platforms for imaging and therapy, their potential as drug carriers is often limited by their typically dense or low-porosity structure. Therefore, achieving effective integration of MnO_2_ into PB NPs enabling excellent MRI performance alongside high drug loading efficiency, and further facilitating synergistic chemo-photothermal therapy for enhanced antitumor efficacy remains a critical challenge to be addressed in current research.

**Scheme 1:**
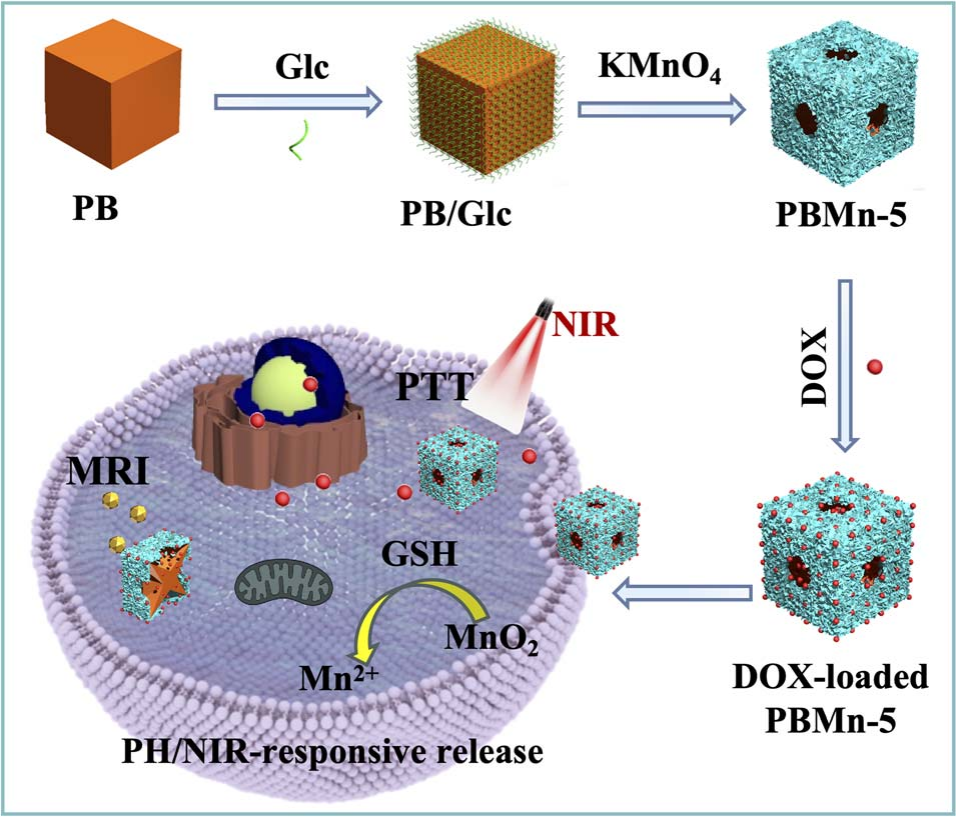
Schematic illustration of the synthetic strategy for PBMn-5 core–shell NPs as pH/NIR dual-responsive drug carriers for MRI and chemo-photothermal combined therapy *in vitro*.

This study reports a facile synergistic strategy involving simultaneous self-etching and coating for the synthesis of PB/MnO_2_ core–shell NPs with an open cage-like structure. As illustrated in Scheme 1, the preparation of these core–shell NPs involves two consecutive steps: first, PB NPs with a regular cubic morphology and smooth surface were synthesized; subsequently, an MnO_2_ shell were grown *in situ* on the PB surface *via* a redox reaction between KMnO_4_ and glucose (Glc).^21^ With prolonged reaction time, manganese species can coordinate with the abundant C≡N groups in the PB framework.^24^ Due to the instability of the Mn–C≡N–Fe structure under these conditions, it gradually decomposes, generating vacancies on the NPs surface. These vacancies are further utilized for loading anticancer drugs. By controlling the reaction time (2, 4, 5, 8, and 15 h) within the same reaction system, the final products (denoted as PBMn-2, PBMn-4, PBMn-5, PBMn-8, and PBMn-15, respectively) were obtained. Finally, drug loaded PBMn-5 core–shell NPs were selected as a responsive drug delivery system for MRI-guided chemo-photothermal combination therapy at the *in vitro* cancer cell level.

## 2 Results and discussion

### 2.1 Synthesis and characterization of PBMn-5 NPs

**Fig. 1:**
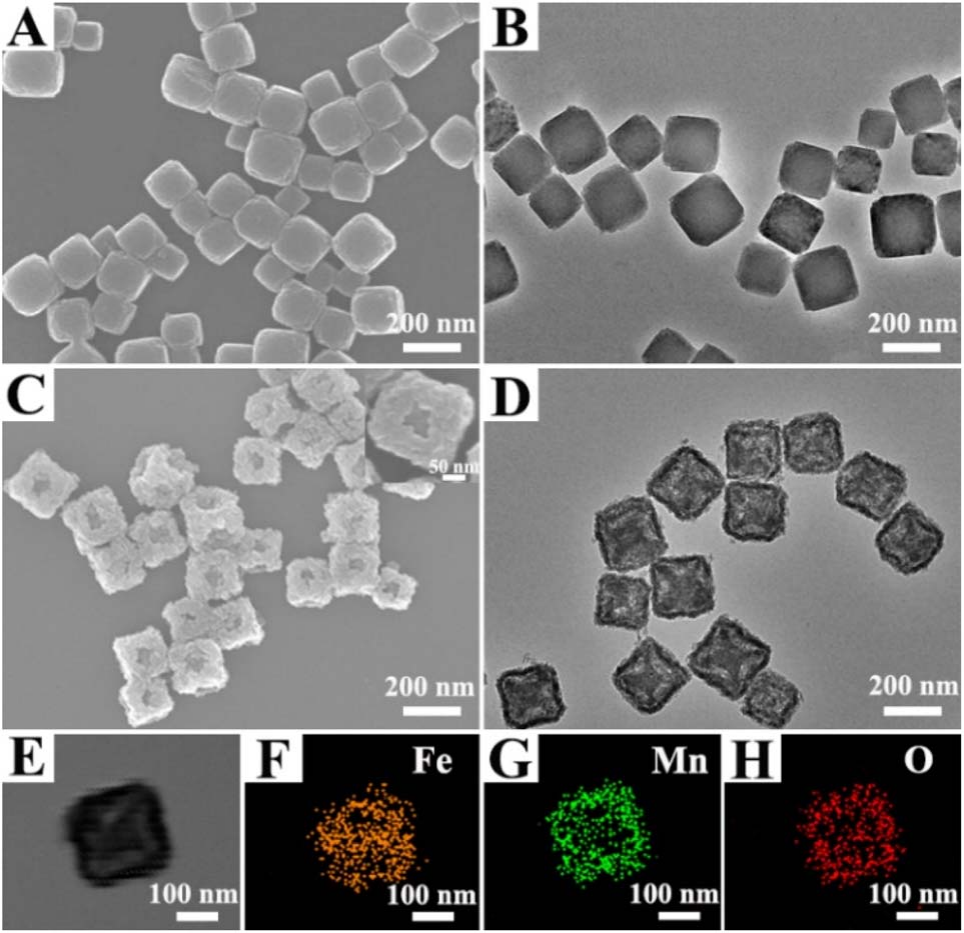
(A) SEM and (B) TEM images of PB NPs. (C) SEM and (D) TEM images of PBMn-5 NPs. (E–H) Elemental mapping images of a single PBMn-5 NP show the distribution of Fe (orange), Mn (green) and O (red) elements.

PB NPs with regular cube shape and smooth surface were prepared according previous work.^25^ Scanning electron microscope (SEM) and transmission electron microscopy (TEM) images showed that the synthesized cubic PB NPs had an average edge length of approximately 192 nm with good dispersity (Fig. 1A and B). Subsequently, an MnO_2_ shell was grown on the PB surface *via* a redox reaction between Glc and KMnO_4_. SEM image revealed that the PBMn-5 NPs exhibited a rough surface with open cavities located predominantly at the large facet centers (Fig. 1C). The pores within the MnO_2_ shell are irregular in shape. This morphology is a direct result of the relatively fast, kinetically controlled growth of the MnO_2_ shell. Meanwhile, TEM image indicated the presence of voids between the MnO_2_ shell and the PB core, with more pronounced etching observed at the facet centers compared to the edges/corners of the PB core (Fig. 1D). The formation of open cavities and the etching of PB are attributed to two correlated steps: the MnO_2_ shell of the PBMn-5 NPs grows progressively from the edges/corners toward the large facet centers, driven by the selective enrichment of Glc at the edge/corner sites.^26^ As the growth rate of the MnO_2_ shell decreases, excess Mn coordinate with the abundant C and N elements in the PB structure, leading to the etching of the PB core^24,27^ To investigate the chemical composition of the PBMn-5 NPs, elemental mapping analysis was performed (Fig. 1E–H). The distribution maps of Fe, Mn, and O confirmed that Mn and O were predominantly located at the outer region of the PBMn-5 NPs. Elemental line scan profiles further supported the presence of a Mn-rich shell, showing higher Mn signal intensity in the outer region compared to Fe (Fig. S1).

The formation of PBMn-5 NPs was further characterized using X-ray powder diffraction (XRD) and X-ray photoelectron spectroscopy (XPS). In the XRD pattern of pure PB (Fig. S2A), several characteristic peaks were observed at 17.5°, 24.7°, 35.3°, 39.6°, 43.5°, 50.7°, 54.0°, 57.2°, 66.1°, and 68.9°, which match unambiguously with the standard reference card for PB. The XRD pattern of the PBMn-5 NPs clearly exhibited the same crystalline diffraction peaks as those of both pure PB and the standard PB card, with no additional crystalline phases detected, indicating that the NPs consist of crystalline PB and amorphous MnO_2_. Furthermore, the presence of manganese and its surface electronic state in the synthesized PBMn-5 NPs were further verified by XPS spectroscopy. As shown in Fig. S3, the XPS spectrum of Mn in the PBMn-5 NPs exhibited binding energies of 642 eV and 653.8 eV, corresponding to Mn 2p_3/2_ and Mn 2p_1/2_, respectively, which is consistent with MnO_2_.^28^ It is well known that nanomaterials with mesoporous structures provide a feasible pathway for drug loading. Therefore, the porosity of the PBMn-5 NPs was determined by N_2_ adsorption–desorption measurements (Fig. S4). The Brunauer–Emmett–Teller (BET) surface area was measured to be 282.13 m^2^ g^−1^, and the total pore volume was 0.44 cm^3^ g^−1^. The pore size distribution profile indicated a mesoporous structure in the NPs, capable of accommodating and releasing drug molecules.

**Fig. 2:**
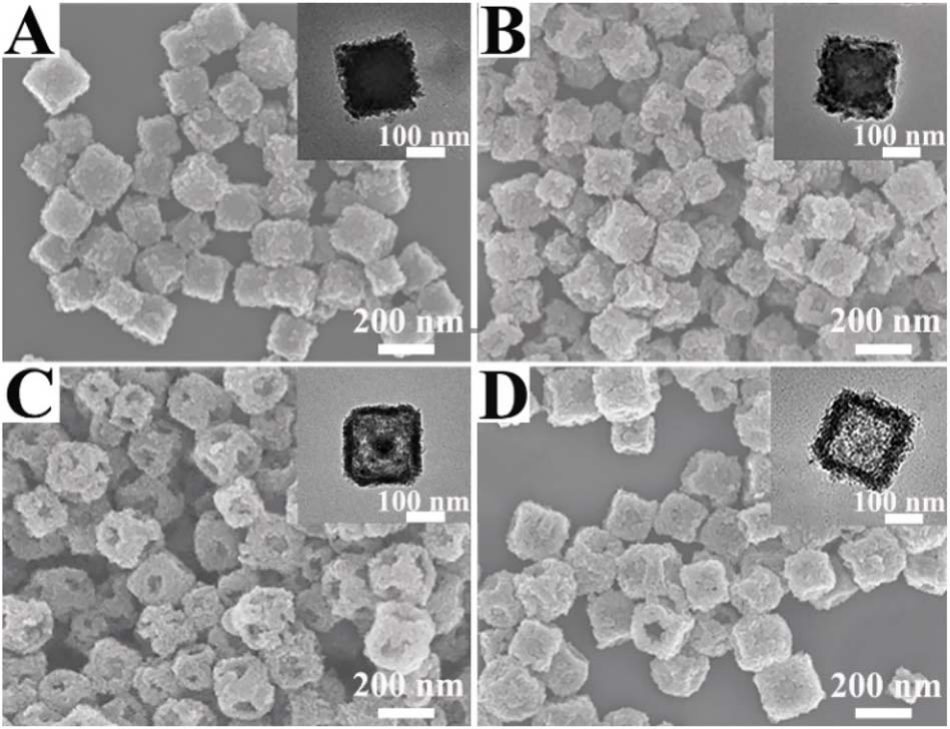
SEM and TEM (inset) images of the NPs with different reaction time: (A) 2 h, (B) 4 h, (C) 8 h, and (D) 15 h.

To elucidate the time dependent morphological evolution of the PBMn-5 NPs, comparative experiments were conducted, and the resulting products were analyzed using SEM and TEM images (Fig. 2). When the reaction time was 2 h, the edges and corners of the PBMn-2 NPs exhibited roughness compared to the centers of the large flat facets (Fig. 2A). When the reaction time reached 4 h, the surface centers of the PBMn-4 NPs exhibited obviously varying degrees of concavity (Fig. 2B). We speculate that this phenomenon may be attributed to the selective and preferential accumulation of glucose at the edges and corners, leading to the preferential growth of the MnO_2_ shell at these sites of the NPs.^29^ When the reaction time was 8 h, an open hole appeared on the surface center of PBMn-8 NPs, and the PB core was etched (Fig. 2C). This phenomenon can be attributed to the ability of Mn to form coordination bonds with the abundant C- and N-based groups in the PB structure. As a result, the redox reaction between Glc and KMnO_4_ not only produced the MnO_2_ shell but also led to structural disruption of PB by excess Mn. At 15 h, the open cavities at the centers of the large flat facets closed, and TEM images clearly revealed the disappearance of the PB core, indicating a hollow structure in the PBMn-15 NPs (Fig. 2D). Furthermore, the disappearance of the PB core was confirmed by comparing the UV-vis absorption spectra (Fig. S5A) and XRD patterns (Fig. S2B) of the nanoparticles obtained at different time points. It was confirmed that the formation of cavities in the NPs originated from the slow growth of the shell from the edges/corners toward the surface centers, coupled with the gradual corrosion of PB over time.

### 2.2 Photothermal effect of PBMn-5 NPs

**Fig. 3:**
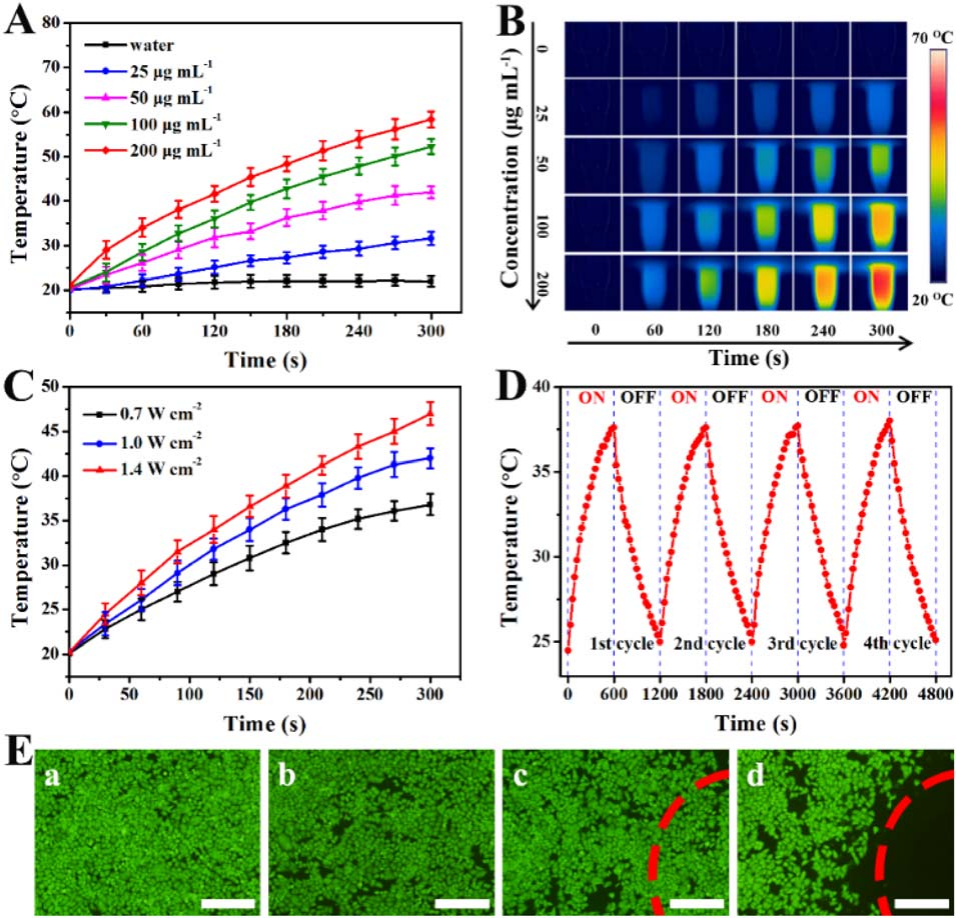
(A) NIR induced temperature increases of PBMn-5 NPs at different concentrations in aqueous solution (808 nm, 1 W cm^−2^). (B) Photothermal images of PBMn-5 NPs solution at different concentrations, and pure water exposed to the 808 nm laser (1 W cm^−2^) recorded at different time intervals (0, 60, 120, 180, 240 and 300 s). (C) NIR induced temperature increase curves at various laser power densities in aqueous solution (50 µg mL^−1^). (D) Temperature monitoring curve of PBMn-5 NPs aqueous suspension (25 µg mL^−1^) during continuous laser on/off cycling. (E) Confocal laser scanning microscopy (CLSM) images of HepG-2 cells with different treatments *via* staining with Calcein-AM: (a) control; (b) PBMn-5 NPs; (c) laser irradiation only; (d) PBMn-5 NPs with laser irradiation (scale bars: 200 µm).

The optical properties of PBMn-5 NPs were investigated, and it was found that even with partial loss of the core groups, they still maintained high absorption in the NIR region (Fig. S5B). Therefore, PBMn-5 NPs are excellent photothermal agents. The temperature changes of PBMn-5 NPs at different concentrations were studied under 808 nm laser irradiation for 300 s (Fig. 3A). The results showed that the solution temperature progressively increased with prolonged irradiation time and higher concentrations of PBMn-5 NPs. After NIR laser irradiation (1.0 W cm^−2^, 5 min), even at a low concentration (25 µg mL^−1^), the solution temperature reached 31.6 °C, while at a concentration of 200 µg mL^−1^, the temperature rose to 58.4 °C. Thus, this approach can easily heat the temperature above 42 °C, leading to irreversible death of cancer cells. The color of the irradiated images captured by the infrared thermal imaging system changed significantly with increasing concentration (Fig. 3B). Additionally, irradiation experiments were conducted on a sample with a concentration of 50 µg mL^−1^ at different laser power densities (Fig. 3C). The temperature increase of the PBMn-5 NPs was positively correlated to the laser output power densities. Importantly, to evaluate photothermal stability, cyclic heating and cooling experiments were performed using PBMn-5 NPs (25 µg mL^−1^) (Fig. 3D). The results indicated that the NPs still maintained excellent photostability after four heating–cooling cycles. The photothermal conversion efficiency (*η*) was calculated using a previously reported method and was approximately 29%, which is comparable with previous reported PB NPs, hollow PB NPs and PB analog (Fig. S6).^30–32^ The calculated photothermal conversion efficiency and outstanding photothermal stability suggest that PBMn-5 NPs have broad application prospects in the field of photothermal therapy.

To further validate the photothermal effect of the synthesized NPs on cancer cells, human hepatocellular carcinoma (HepG-2) cells were seeded in a 96-well plate and incubated with 40 µg per mL PBMn-5 NPs, followed by irradiation with an 808 nm NIR laser at a power density of 1.0 W cm^−2^ for 5 min. The treated cells were stained with Calcein AM, a fluorescent dye that permeates live cells, to observe cell viability. As shown in Fig. 3E, extensive cell death was observed in HepG-2 cells treated with PBMn-5 NPs and subjected to NIR laser irradiation. In contrast, most cells outside the irradiated area exhibited strong green fluorescence from Calcein AM, indicating that these cells remained alive. In comparison, no significant cell death was observed in the negative control group (without any treatment), the group treated with PBMn-5 NPs alone (without laser irradiation), or the group receiving laser irradiation only (in the absence of NPs). These results collectively demonstrated that PBMn-5 NPs can effectively ablate cancer cells under NIR laser irradiation, while exhibiting low cytotoxicity and no significant cell death in the absence of irradiation. This further confirms the potential of PBMn-5 NPs for application in PTT.

### 2.3 Drug loading, pH/NIR dual-responsive controlled release and cytotoxicity assays *in vitro*

**Fig. 4:**
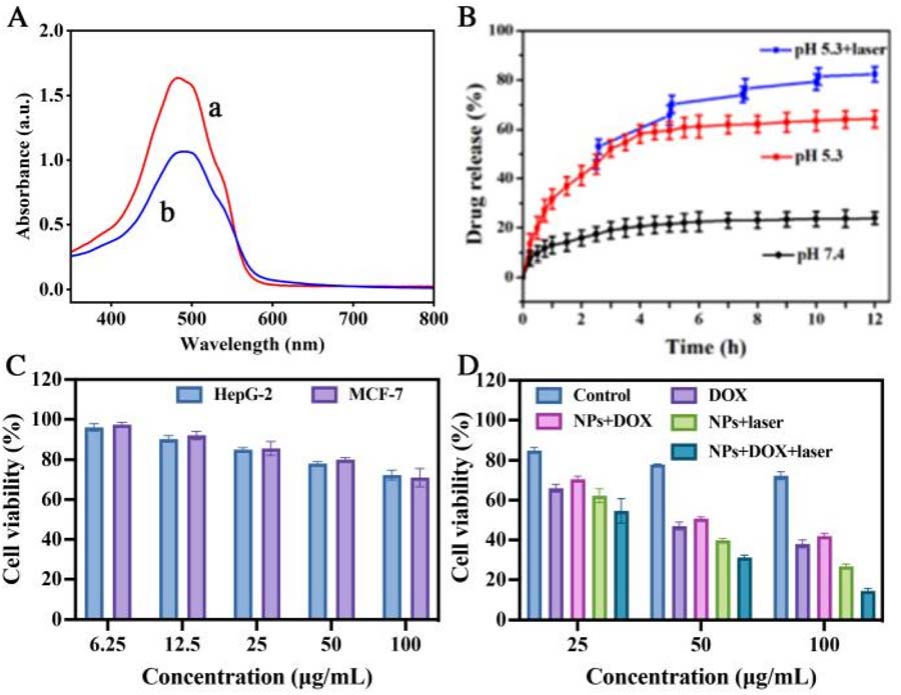
(A) UV-vis absorption spectra of original DOX aqueous solution (a) and the residual DOX content in the supernatant after interaction with PBMn-5 NPs (b). (B) DOX-release profiles of DOX from DOX-loaded PBMn-5 NPs in PBS buffer: pH = 7.4, 5.3 and 5.3 with periodic laser ON/OFF irradiation (808 nm, 1.0 W cm^−2^). (C) Cell viabilities of HepG-2 and MCF-7 cells treated with different dosages of PBMn-5 NPs. (D) Cytotoxicity assays of HepG-2 cells incubated with different treatments.

We selected doxorubicin (DOX) as the model antitumor drug and verified the high loading capacity and release properties of PBMn-5 NPs for DOX using UV-vis absorption spectroscopy. As shown in Fig. 4A, the UV-vis absorption spectra of the DOX aqueous solution and the supernatant of DOX loaded PBMn-5 NPs showed obvious decrease in the characteristic drug absorption peak. The calculated drug loading capacity reached approximately 0.36 mg per mg of PBMn-5 NPs. Fig. 4B showed the *in vitro* drug release behavior of PBMn-5 NPs in phosphate buffered saline (PBS) at different pH values (pH = 5.3 and 7.4), with and without NIR laser irradiation (1.0 W cm^−2^). The results demonstrated pH/NIR dual responsive release characteristics. In PBS at pH 7.4, only 23.9% of DOX was released from the NPs within 12 h, indicating good stability of the drug loaded system under physiological conditions, which is beneficial for maintaining structural integrity during blood circulation. In contrast, under acidic conditions (pH = 5.3), the cumulative release of DOX reached 64.3% within the same period. When NIR laser irradiation was applied at pH 5.3, the release amount further increased to 82.3%, indicating that NIR irradiation significantly enhances drug release in an acidic environment. Moreover, the structure and absorption spectra of PBMn-5 were detected after different treatment. The result shown that the nanostructure keep stable after NIR laser irradiation, but damaged in acidic or GSH solution (Fig. S7 and S8). This release behavior can be attributed to the protonation of the amino groups in DOX molecules under acidic conditions, which weakens the interaction between DOX and PBMn-5 NPs.^33^ NIR laser irradiation promoting faster diffusion of DOX from NPs, and further reduces the binding force, leading to more efficient and thorough drug release. Moreover, the nanostructure damaged to a certain extent, inducing the release of DOX. Given that the tumor microenvironment is generally weakly acidic, these DOX loaded PBMn-5 NPs with pH/NIR dual responsive drug release characteristics demonstrate potential as an ideal antitumor drug delivery system.

To further explore the therapeutic effect of chemo-photothermal combined therapy, HepG-2 cells were treated with PBMn-5 NPs first to estimate the cell viability of pure NPs. As present in Fig. 4C, there is almost no cytotoxicity at lower concentration, but slight cytotoxicity at higher concentration. The same result was observed in MCF-7 cells. Upon incubation of HepG-2 cells with free DOX, DOX-loaded PBMn-5 NPs or PBMn-5 NPs followed by NIR laser irradiation (1.0 W cm^−2^, 5 min), the observed reduction in cell viability confirmed the distinct therapeutic effects of chemotherapy and photothermal therapy, respectively. Compared to the NPs + DOX group, the free DOX group exhibited slightly stronger cytotoxicity. This difference can be attributed to the slow and sustained release profile of DOX from our PBMn-5 nanocarriers. The most significant cell death occurred in the group treated with DOX-loaded PBMn-5 nanoparticles under NIR laser irradiation. At a concentration of 100 µg mL^−1^, the viability of HepG-2 cells was reduced to less than 20% (Fig. 4D). These results demonstrated that DOX loaded PBMn-5 NPs under NIR laser irradiation can serve as a highly effective combined chemo-photothermal therapeutic agent, significantly enhancing the killing effect on cancer cells.

### 2.4 Confocal laser scanning microscopy studies of the cellular uptake efficiency

**Fig. 5:**
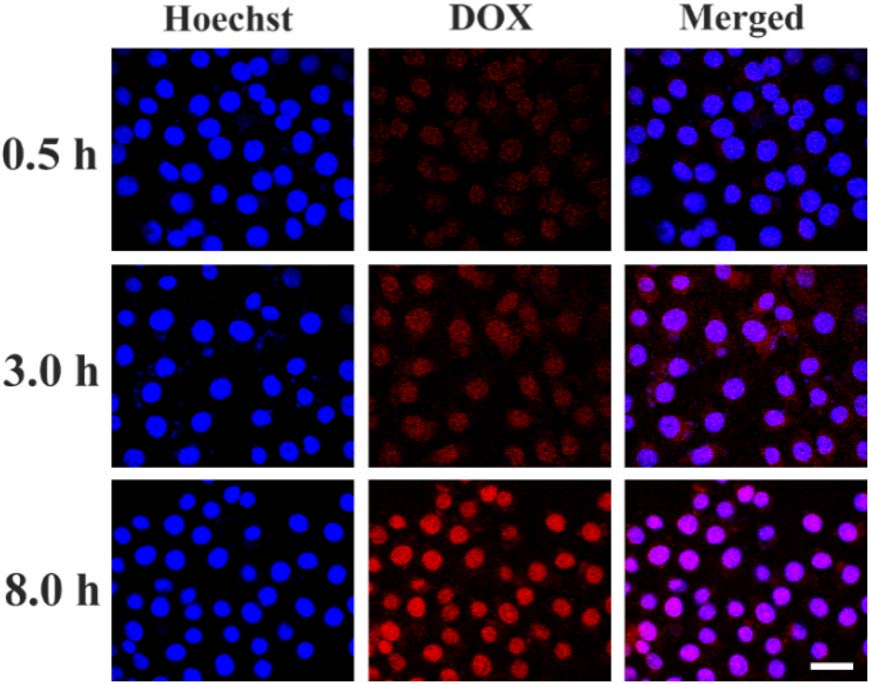
CLSM images of HepG-2 cells incubated with DOX-loaded PBMn-5 NPs for 0.5, 3, and 8 h (scale bar: 50 µm).

We further investigated the cellular uptake and distribution behavior of DOX loaded PBMn-5 NPs in HepG-2 cells. DOX loaded PBMn-5 NPs were incubated with HepG-2 cells *in vitro* at 37 °C, and the nuclei were stained with Hoechst 33342. CLSM images were acquired at different time points. As shown in Fig. 5, the strongest red fluorescence signal was observed after 8 h of incubation. The merged images exhibited a purple color, resulting from the overlap of blue nuclear staining and red DOX fluorescence, indicating the most amount of DOX within cells and was located in nuclei region. Images merged over time from low to high intensity also revealed the process of DOX entering the nucleus, indicating that the DOX loaded PBMn-5 NPs can effectively deliver the drug into the cells.

### 2.5 MRI signal enhancement of PBMn-5 NPs *in vitro*

**Fig. 6:**
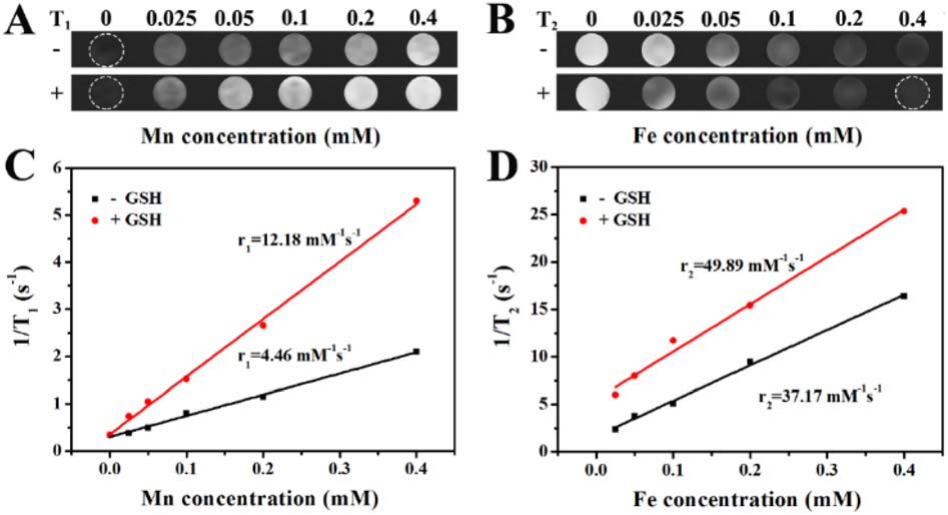
(A) *T*_1_-Weighted MR images of PBMn-5 NPs at different conditions recorded. (B) *T*_2_-Weighted MR images of PBMn-5 NPs at different conditions recorded. (C and D) Relaxation rate 1/*T*_1_ and 1/*T*_2_ as a function of the concentration of PBMn-5 NPs before and after adding GSH (15 mM).

To evaluate the performance of PBMn-5 NPs as magnetic resonance imaging (MRI) contrast agents, we measured their *T*_1_-weighted and *T*_2_-weighted MRI signals under different conditions. Considering that the expression level of GSH in cancer cells is significantly higher than that in normal tissues, and that MnO_2_ can react with GSH in the tumor microenvironment to generate Mn^2+^ (Fig. S9). After treated with GSH, the UV-vis spectra before and after treatment changed (Fig. S7 and S9A). We observe that the amount of NPs decreased, and resident NPs were perform by TEM images (Fig. S8D), which show that the structure destroyed.^21^ We further analyzed the changes in *T*_1_-weighted and *T*_2_-weighted MRI signals of PBMn-5 NPs in the presence of GSH. After reduction treatment with GSH, the PBMn-5 NPs exhibited more significant signal enhancement in both *T*_1_-weighted and *T*_2_-weighted images (Fig. 6A and B). Fig. 6C and D showed the relationship between NPs concentration and longitudinal relativity (*r*_1_) and transverse relativity (*r*_2_) under different treatment conditions, respectively. After reduction with 15 mM GSH, the *r*_1_ and *r*_2_ values reached 12.18 mM^−1^ s^−1^ and 49.89 mM^−1^ s^−1^, respectively. These results indicated that PBMn-5 NPs can undergo a responsive reaction in the high intracellular GSH microenvironment and function as an efficient *T*_1_/*T*_2_ dual-mode MRI contrast agent.

## 3 Conclusion

In summary, this study developed a biodegradable PBMn-5 NPs that integrates photothermal therapy, chemotherapy, and magnetic resonance imaging. A simultaneous self-etching process during MnO_2_ coating preserved the cubic structure of PB and created cavities for drug loading. The NPs combined strong NIR absorption with ultrahigh drug loading capacity, enabling synergistic chemo-photothermal therapy with significantly enhanced antitumor efficacy. Furthermore, MnO_2_ could enhance imaging contrast by depleting intracellular GSH. Experiments demonstrated that PBMn-5 serves as a *T*_1_/*T*_2_ dual-mode MRI contrast agent, exhibiting significantly improved relaxivity after reaction with GSH. This easily constructed theranostic nanoplatform offers a new clinical translation pathway for precision cancer treatment.

## 4 Experimental section

### 4.1 Materials

Potassium hexacyanoferrate(iii), polyvinylpyrrolidone (PVP, K30) and doxorubicin hydrochloride (DOX) were procured from Sigma (USA). Hydrochloric acid (HCl), glutathione (GSH) and glucose (Glc) were procured from Beijing Chemical Works. KMnO_4_ was procured from Sinopharm Chemical Reagent Beijing Co., Ltd. Deionized water was used in all experiments.

### 4.2 Synthesis of PB NPs

Briefly, 3 g of PVP and 0.264 g of potassium ferricyanide (K_3_[Fe(CN)_6_]) were added to 80 mL of 0.01 M hydrochloric acid solution. The mixture was stirred with a magnetic stirrer until completely dissolved, yielding a clear yellow solution. This solution was then reacted at a constant temperature of 80 °C for 24 h. During the reaction, the color of the solution gradually changed from yellow to green, and finally to blue. After the reaction was completed, the solution was cooled, and PB NPs were collected by centrifugation at 8000 rpm for 20 min. The collected nanoparticles were washed twice with ethanol and twice with ultrapure water, respectively. Finally, the resulting NPs were dispersed in 20 mL of ultrapure water for further use.

### 4.3 Synthesis of PBMn-5 NPs

At room temperature, a manganese dioxide shell was synthesized *in situ* on the surface of PB NPs *via* a redox reaction between potassium permanganate and glucose. The detailed procedure is as follows: first, Glc (0.02 g) and PB nanoparticle dispersion (4 mL) were added to 16 mL of ultrapure water. The mixture was stirred continuously at room temperature for at least 1 hour to allow sufficient adsorption of glucose onto the PB NPs surfaces. Subsequently, 0.015 g of KMnO_4_ was added to the solution, and the reaction was allowed to proceed under stirring for 5 h. After the reaction, the precipitate was collected by centrifugation and washed twice with ultrapure water and twice with ethanol, respectively. Finally, the obtained product was dried at 50 °C for 12 h to yield PBMn-5 NPs, which can be used directly in subsequent experiments.

### 4.4 Cell culture

Human hepatocellular carcinoma (HepG-2) cells and Michigan cancer foundation-7 (MCF-7) were purchased from procell and cultured as a monolayer in Dulbecco's Modified Eagle's Medium (DMEM) supplemented with 10% fetal bovine serum, and maintained at 37 °C in a humidified atmosphere of 95% air and 5% CO_2_.

### 4.5 Photothermal therapy of PBMn-5 NPs

The photothermal performance of PBMn-5 NPs was evaluated in an aqueous system. Dispensions of PBMn-5 NPs at various concentrations (0, 25, 50, 100, and 200 µg mL^−1^) were exposed to an 808 nm NIR laser (1.0 W cm^−2^) for 300 s, and the temperature changes were recorded every 30 s using an infrared thermal imager. To investigate the photothermal conversion stability, four cycles of on/off laser irradiation were performed, with each cycle consisting of 10 min of irradiation followed by 10 min of natural cooling. At the cellular level, the photothermal effect was assessed by Calcein-AM staining. HepG-2 cells were seeded in a 96-well plate at a density of 2.5 × 10^4^ cells per well and cultured for 24 h. The cells were then divided into four groups: group 1 served as a control with DMEM medium only; group 2 was incubated with PBMn-5 nanoparticles (40 µg mL^−1^); group 3 received near-infrared laser irradiation only (1.0 W cm^−2^, 5 min); group 4 was treated with both PBMn-5 NPs (40 µg mL^−1^) and near-infrared laser irradiation (1.0 W cm^−2^, 5 min). After all treatments, the cells were stained with Calcein-AM and analyzed.

### 4.6 Calculation of the photothermal conversion efficiency (*η*)

The photothermal conversion efficiency (*η*) was determined by continuously irradiating a dispersion of PBMn-5 NPs (25 µg mL^−1^) with an 808 nm NIR laser (1.0 W cm^−2^) and recording the temperature change over time until a steady state was reached. After turning off the laser, the cooling process of the aqueous suspension was monitored to quantify the rate of heat dissipation from the system to the environment. The photothermal conversion efficiency (*η*) was calculated according to a previously reported method.1
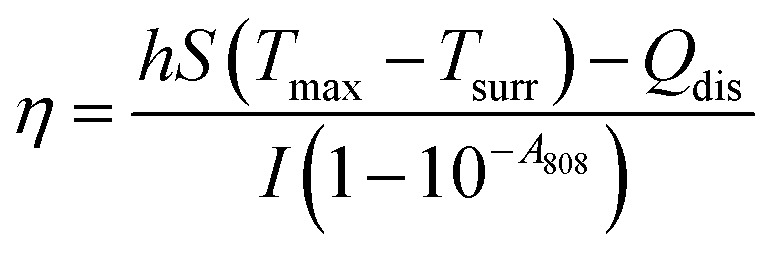
Here, *h* is the heat transfer coefficient, *S* is the heat transfer surface area of the sample cell, *T*_max_ is the maximum equilibrium temperature reached by the system, *T*_surr_ is the ambient temperature, *Q*_dis_ represents the heat generated by the background absorption of the solvent, *I* is the incident laser power density (1 W cm^−2^), and *A*_808_ is the absorbance of PBMn-5 nanoparticles at 808 nm. The value of *hS* was calculated using the following eqn (2):2
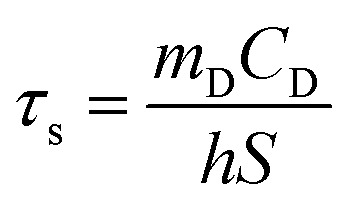
where *τ*_s_ is the time constant of the sample system, and *m*_D_ and *C*_D_ represent the mass and heat capacity of the solvent (water), respectively.

### 4.7 Drug loading and release *in vitro*

The DOX-loaded NPs were prepared by dissolving 1.2 mg of DOX in 1 mL of ultrapure water, followed by mixing with 1.2 mg of PBMn-5 NPs under light-protected stirring at room temperature for 24 h. Afterward, unadsorbed DOX was removed by centrifugation, and the product was washed three times with ultrapure water to obtain the DOX-loaded PBMn-5 NPs. The initial DOX content and the residual DOX content in the supernatant were determined by measuring the ultraviolet-visible absorption. The drug loading capacity (LC) of DOX can be calculated using eqn (3):3
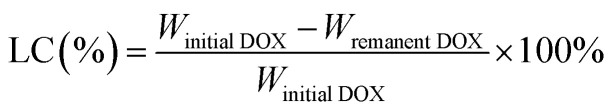
where *W*_initial_ represents the mass of DOX in the initial solution, and *W*_remanent DOX_ denotes the mass of residual DOX in the supernatant.

The drug release behavior of DOX-loaded PBMn-5 NPs was evaluated using a dialysis bag diffusion method in PBS at 37 °C with pH values of 5.3 and 7.4. The DOX loaded PBMn-5 NPs were divided into three equal portions. Two portions were dispersed in PBS at pH 5.3 and pH 7.4, respectively. These two samples were placed into pre-treated semipermeable dialysis bags, which were then immersed in containers with 5 mL of PBS at corresponding pH values (37 °C) under gentle shaking. At predetermined time intervals, the amount of DOX released from the dialysis bags into the external buffer was measured at 480 nm using a UV-vis spectrophotometer. To investigate the enhancing effect of laser irradiation on drug release, the third sample (in pH 5.3 PBS) was exposed to an 808 nm laser at a power density of 1 W cm^−2^ for 5 min, and its release profile was simultaneously analyzed.

### 4.8 Cytotoxicity assays *in vitro*

3-(4,5-Dimethyl-thiazol-2-yl)-2,5 diphenyltetrazolium bromide (MTT) assays were used to evaluate the PBMn-5 NPs doses on the toxicity of the cells. HepG-2 and MCF-7 cells were seeded into 96-well plates at a density of 2.5 × 10^4^ cells per well and incubated for 24 h. The cells were treated with various concentrations of PBMn-5 NPs (6.25, 12.5, 25, 50, and 100 µg mL^−1^) in fresh DMEM for 24 h. In another plate, the PBMn-5 NPs and DOX-loaded PBMn-5 NPs with different concentrations were added into HepG-2 cells with NIR laser irradiation (1.0 W cm^−2^, 5 min). Treated cells were mixed with DMEM containing 10 µL MTT and further incubated for 4 h at 37 °C. After incubation, the MTT assay was performed to quantitatively measure the relative cell viability. Cell viability was determined by eqn (4):4
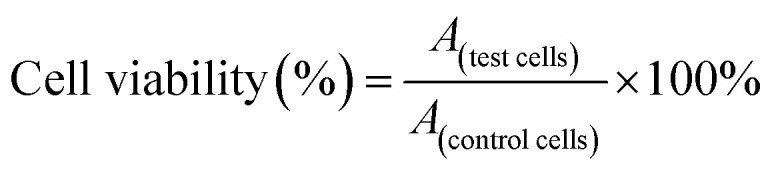


### 4.9 Cell uptake

The HepG-2 cells were seeded in 24-well plates with a clean cover-slip per well and incubated with 15 µg mL^−1^ of DOX-loaded PBMn-5 NPs for 0.5, 3.0, and 8.0 h in the environment of 5% CO_2_ at 37 °C. After incubation, the cells were washed by PBS for three times to remove the excess NPs and the dead cells. Subsequently, HepG-2 cells were stained with hoechst 33 342. The cover-slips were mounted onto a glass microscope slide and the cells were observed by Confocal Laser Scanning Microscopy.

### 4.10 MRI signal enhancement of PBMn-5 NPs *in vitro*

Firstly, aqueous suspensions of PBMn-5 NPs at various concentrations were prepared, and their corresponding longitudinal relaxation time (*T*_1_) and transverse relaxation time (*T*_2_) were measured. All *in vitro* magnetic resonance imaging experiments were performed on a 1.2 T MRI system. In addition, following the same experimental procedure, the regulatory effect of GSH on the magnetic resonance relaxation signals of PBMn-5 NPs was systematically investigated.

## Conflicts of interest

The authors declare no conflict of interest.

## Supplementary Material

RA-016-D5RA07493B-s001

## Data Availability

The data underlying this study are available in the published article and its supplementary information (SI). Supplementary information: experimental results including XRD spectra, UV-vis absorption spectra, TEM images after different treatments, N_2_ adsorption–desorption isotherms, XPS spectrum. See DOI: https://doi.org/10.1039/d5ra07493b.
