# Correction: Biodegradable Prussian blue/manganese dioxide core–shell nanoparticles with open cages for imaging-guided chemo-photothermal combined therapy of cancer cells

**DOI:** 10.1039/d6ra90046a

**Published:** 2026-05-26

**Authors:** Ying Gao, Jinbo Xue, Yuebo Yang, Dongxiao Bian, Luyao Liu, Ming Zhu, Tao Yang, Le Liu

**Affiliations:** a Department of Stomatology, The 964th Hospital of the Chinese People’s Liberation Army Joint Logistics Support Force Changchun Jilin China 1595765865@qq.com 1350288208@qq.com; b Faculty of Education, The University of Hong Kong Hong Kong China

## Abstract

Correction for ‘Biodegradable Prussian blue/manganese dioxide core–shell nanoparticles with open cages for imaging-guided chemo-photothermal combined therapy of cancer cells’ by Ying Gao *et al.*, *RSC Adv.*, 2026, **16**, 10372–10379, https://doi.org/10.1039/D5RA07493B.

The authors regret that Fig. 3E and 5 in the original article were included by mistake. The images were obtained from different batches of cells and various experiments and did not correspond to the experimental system described in the manuscript.

All formal experiments in this study were performed with low-passage HepG-2 cells obtained from the supplier described in the Experimental section of the article. The cells initially used to capture images for Fig. 3E and 5 were also HepG-2 cells, but they were sourced from a different supplier than the one stated in the manuscript. In addition, these cells had been cultured for an extended period. The authors observed morphological changes compared with the early-passage HepG-2 cells. Considering the long-term culture, the authors cannot completely rule out potential cell contamination or phenotypic drift during maintenance. Whilst sorting raw experimental images, the micrographs from these long-term cultured cells were mistakenly included in the manuscript.

Fig. 3E and 5 have been replaced accordingly. The correct images are provided below.

All other data in the manuscript are confirmed to have been obtained using the correct experimental system, which is consistent with the information described in the Experimental section of the article. The authors declare that this figure replacement does not affect any experimental results, data interpretation or conclusions of the study.

An independent expert has viewed the corrected images and has concluded that they are consistent with the discussions and conclusions presented.

**Fig. 1:**
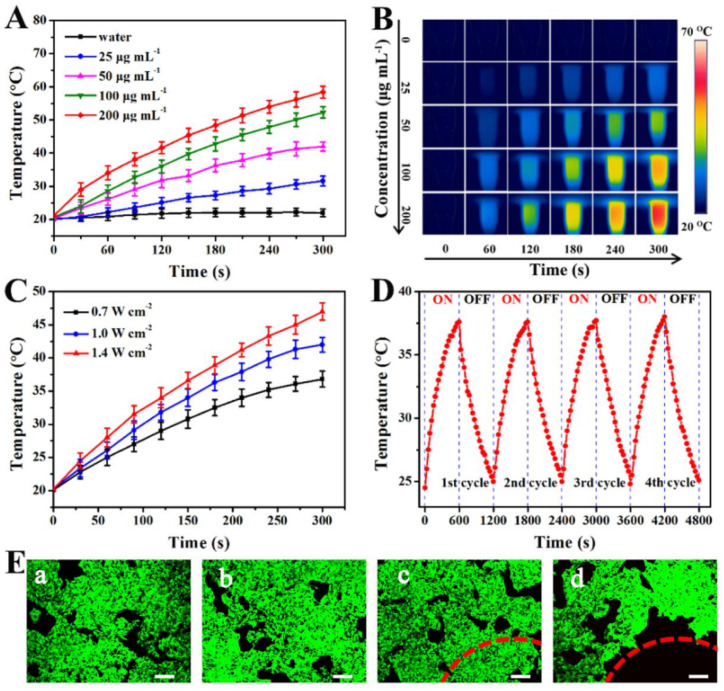
(A) NIR induced temperature increases of PBMn-5 NPs at different concentrations in aqueous solution (808 nm, 1 W cm^−2^). (B) Photothermal images of PBMn-5 NPs solution at different concentrations, and pure water exposed to the 808 nm laser (1 W cm^−2^) recorded at different time intervals (0, 60, 120, 180, 240 and 300 s). (C) NIR induced temperature increase curves at various laser power densities in aqueous solution (50 µg mL^−1^). (D) Temperature monitoring curve of PBMn-5 NPs aqueous suspension (25 µg mL^−1^) during continuous laser on/off cycling. (E) Confocal laser scanning microscopy (CLSM) images of HepG-2 cells with different treatments *via* staining with Calcein-AM: (a) control; (b) PBMn-5 NPs; (c) laser irradiation only; (d) PBMn-5 NPs with laser irradiation (scale bars: 200 µm).

**Fig. 2:**
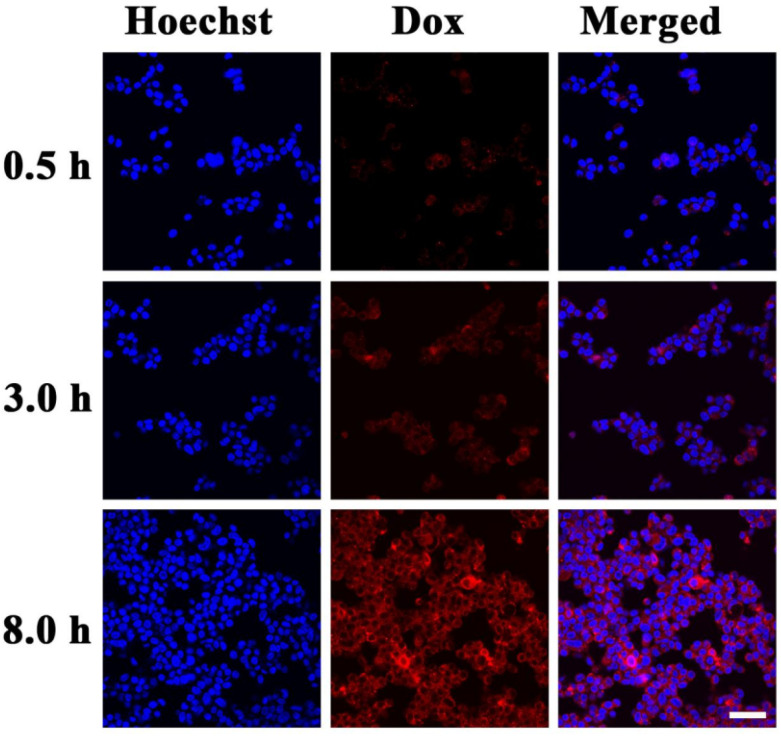
CLSM images of HepG-2 cells incubated with DOX-loaded PBMn-5 NPs for 0.5, 3, and 8 h (scale bar: 50 µm).

The Royal Society of Chemistry apologises for these errors and any consequent inconvenience to authors and readers.

