# Supplementary material for: Biodegradable Prussian blue/manganese dioxide core–shell nanoparticles with open cages for imaging-guided chemo-photothermal combined therapy of cancer cells
Source: RSC Adv. 2026 Feb 23;16(12):10372–9. doi: 10.1039/d5ra07493b (PMC12926748; doi:10.1039/d5ra07493b)
Supplement: RA-016-D5RA07493B-s001 [file RA-016-D5RA07493B-s001.pdf]

Supplementary Information (SI) for RSC Advances.
This journal is © The Royal Society of Chemistry 2026

Supporting information

Biodegradable prussian blue/manganese dioxide core-shell nanoparticles with open cages for imaging-guided chemo-photothermal combined therapy of cancer cells

Ying Gao[a,1], Jinbo Xue[a,1], Yuebo Yang[b], Dongxiao Bian[a], Luyao Liu[a], Ming Zhu[a], Tao Yang[a,*], Le Liu[a,*]

[1]Y.G. and J.X. contributed equally to this work.

[a.]Department of Stomatology, NO. 964 hospital of the chinese people's liberation army joint logistics support force, Jilin 130021, PR China, E-mail: 1595765865@qq.com, 1350288208@qq.com

[b.]National University of Singapore, 21 Lower Kent Ridge Rd, 119077, Singapore

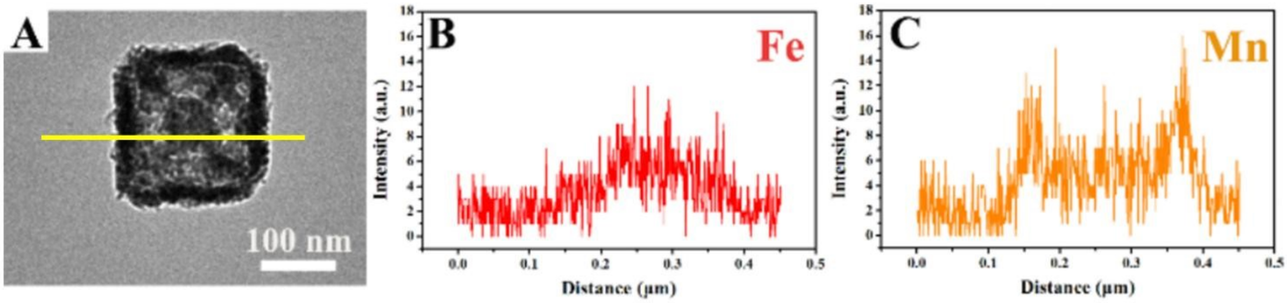

Fig S1. (A) TEM image of a single PBMn-5 NPs. (B-C) Elemental line scan analysis of PBMn-5 NPs.

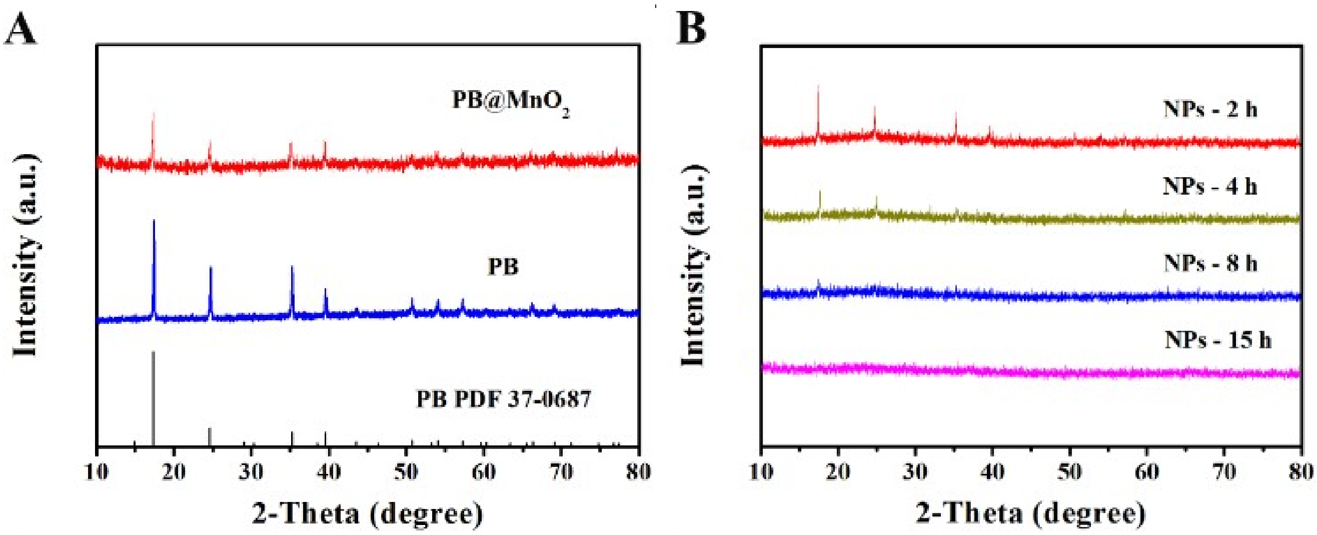

Fig S2. (A) XRD patterns of PB and PBMn-5 NPs. (B) XRD patterns of NPs with different reaction time (2, 4, 8, and 15 h).

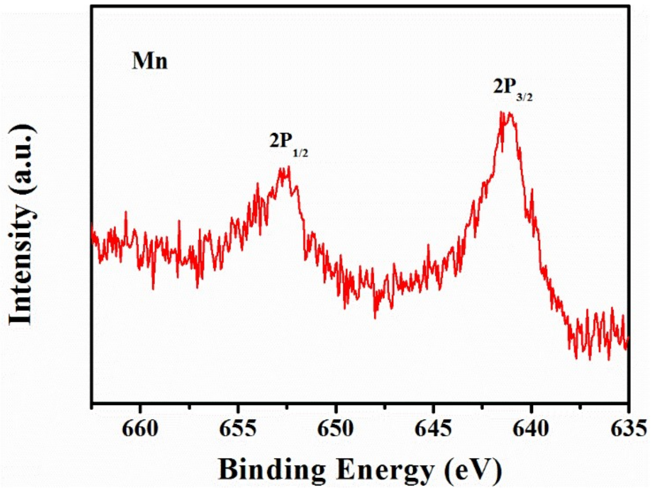

Fig S3. XPS spectrum of Mn elements in PBMn-5 NPs.

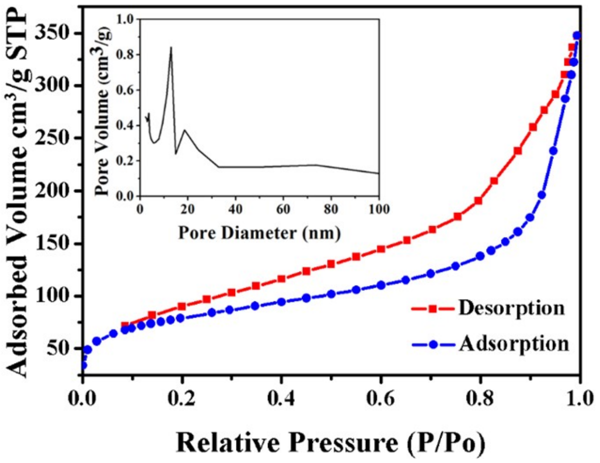

Fig S4. N_2 adsorption–desorption isotherms and pore-size distribution curve (inset) of the PBMn-5 NPs.

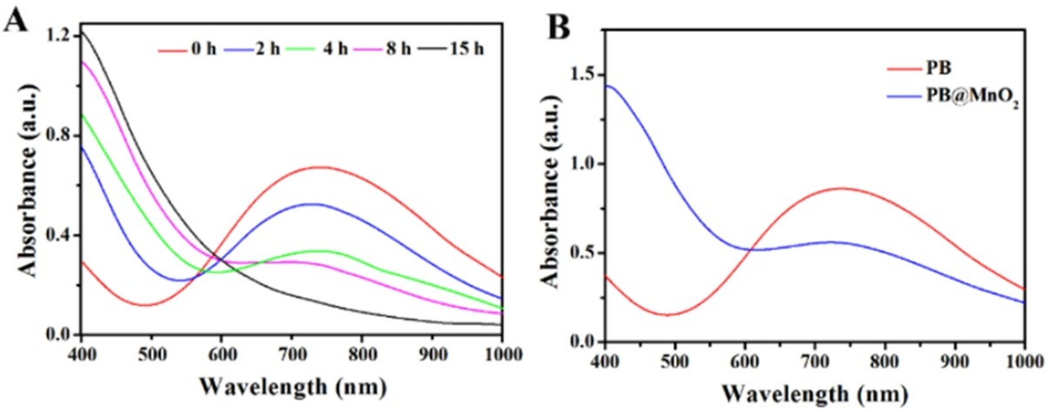

Fig S5. (A) UV-Vis absorption spectra of NPs with different reaction time (0, 2, 4, 8, and 15 h). (B) UV-Vis absorption spectra of PB NPs and PBMn-5 NPs.

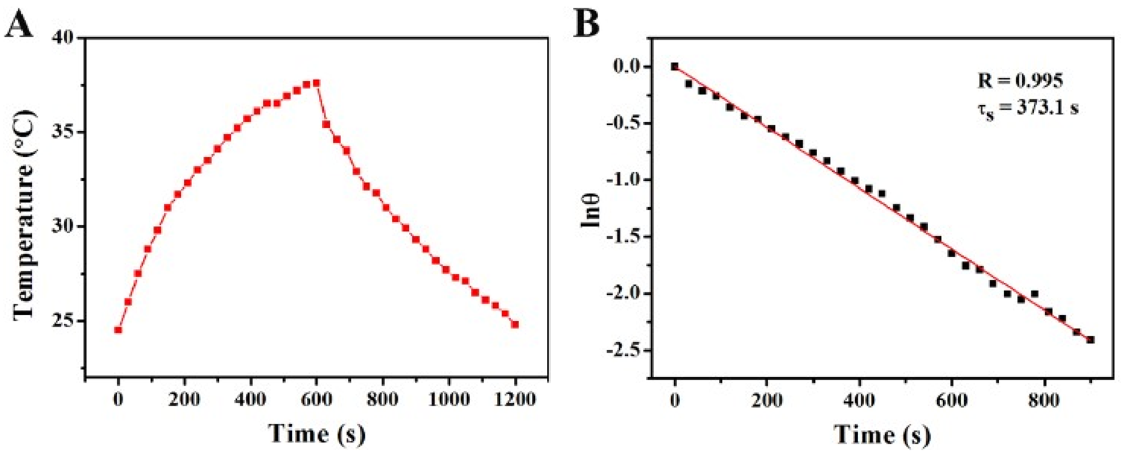

Fig S6. (A) Photothermal response of the aqueous dispersion of PBMn-5 NPs under NIR laser (808 nm, 1.0 W cm^{-2}) irradiation for 10 min. (B) Linear time date versus-lnθ obtained from the cooling period.

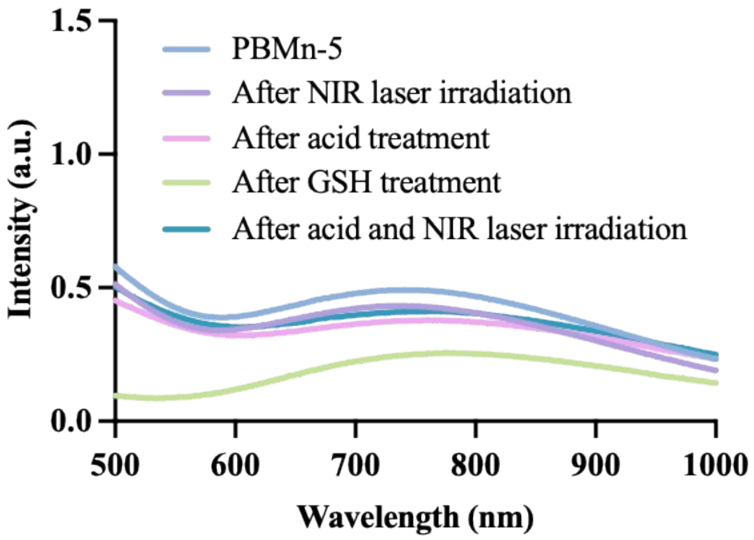

Fig S7. UV-Vis absorption spectra of PBMn-5 NPs after different treatment.

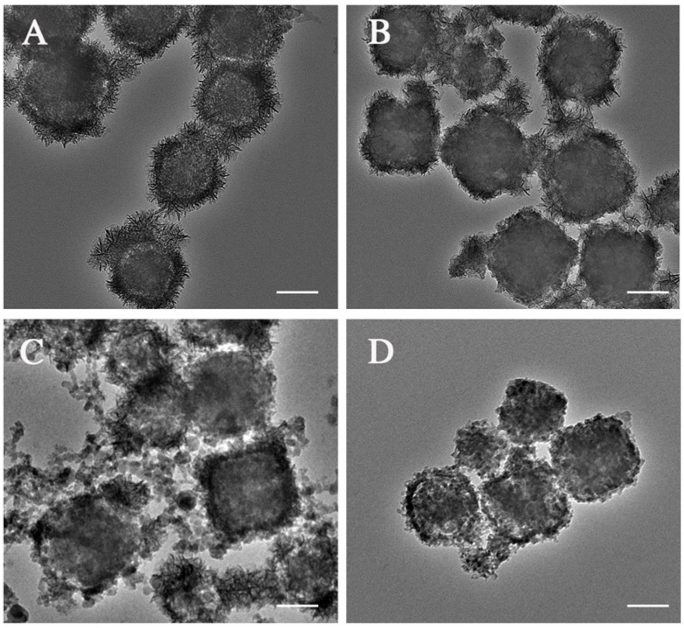

Fig S8. TEM images of PBMn-5 NPs (A) before and after treated with (B) NIR laser irradiation, (C) acidic PBS, and (D) GSH. The scale bar is 100 nm.

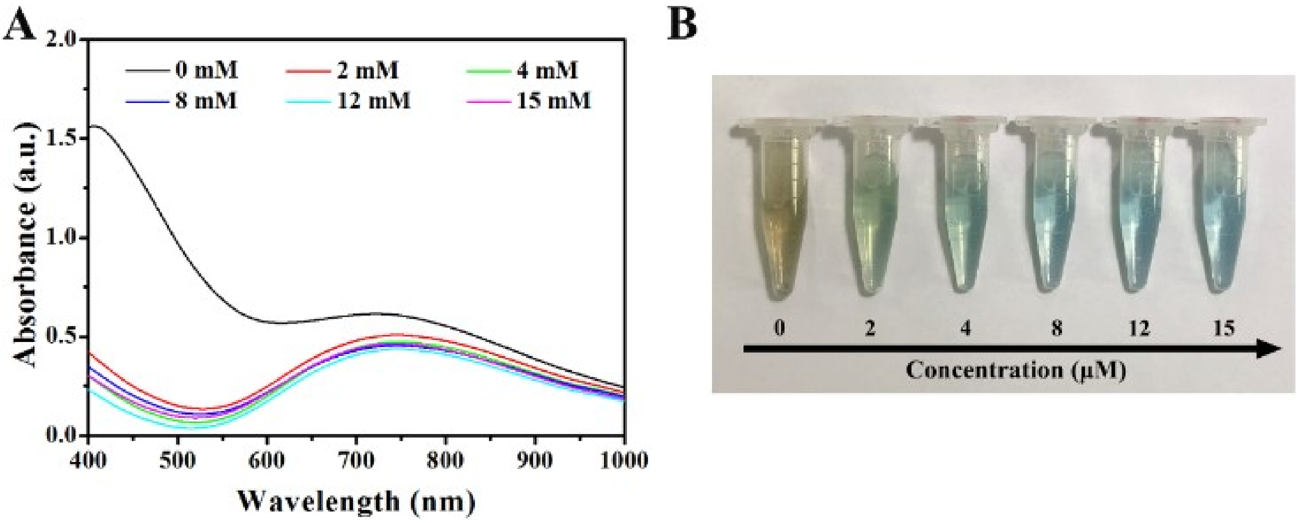

Fig S9. (A) UV-vis absorption spectra of PBMn-5 NPs after reaction with increased concentration of GSH. (B) Digital images of PBMn-5 NPs (0.1 mg mL^{-1}) after reaction with increased concentration of GSH.
